# Assessing socioeconomic health care utilization inequity in Israel: impact of alternative approaches to morbidity adjustment

**DOI:** 10.1186/1471-2458-11-609

**Published:** 2011-08-01

**Authors:** Efrat Shadmi, Ran D Balicer, Karen Kinder, Chad Abrams, Jonathan P Weiner

**Affiliations:** 1 Faculty of Social Welfare and Health Sciences, University of Haifa, Mount Carmel 31905, Israel; 2 2 Clalit Research Institute and Health Policy Planning Department, Chief Physician's Office, Clalit Health Services, 101 Arlozorov St, Tel Aviv 62098, Israel; 3 Epidemiology Department, Faculty of Health Sciences, Ben-Gurion University, Beer-Sheva 84105, Israel; 4 Bloomberg School of Public Health, Johns Hopkins University, Baltimore, MD 21205, USA

## Abstract

**Background**

The ability to accurately detect differential resource use between persons of different socioeconomic status relies on the accuracy of health-needs adjustment measures. This study tests different approaches to morbidity adjustment in explanation of health care utilization inequity.

**Methods**

A representative sample was selected of 10 percent (~270,000) adult enrolees of Clalit Health Services, Israel's largest health care organization. The Johns-Hopkins University Adjusted Clinical Groups^® ^were used to assess each person's overall morbidity burden based on one year's (2009) diagnostic information. The odds of above average health care resource use (primary care visits, specialty visits, diagnostic tests, or hospitalizations) were tested using multivariate logistic regression models, separately adjusting for levels of health-need using data on age and gender, comorbidity (using the Charlson Comorbidity Index), or morbidity burden (using the Adjusted Clinical Groups). Model fit was assessed using tests of the Area Under the Receiver Operating Characteristics Curve and the Akaike Information Criteria.

**Results**

Low socioeconomic status was associated with higher morbidity burden (1.5-fold difference). Adjusting for health needs using age and gender or the Charlson index, persons of low socioeconomic status had greater odds of above average resource use for all types of services examined (primary care and specialist visits, diagnostic tests, or hospitalizations). In contrast, after adjustment for overall morbidity burden (using Adjusted Clinical Groups), low socioeconomic status was no longer associated with greater odds of specialty care or diagnostic tests (OR: 0.95, CI: 0.94-0.99; and OR: 0.91, CI: 0.86-0.96, for specialty visits and diagnostic respectively). Tests of model fit showed that adjustment using the comprehensive morbidity burden measure provided a better fit than age and gender or the Charlson Index.

**Conclusions**

Identification of socioeconomic differences in health care utilization is an important step in disparity reduction efforts. Adjustment for health-needs using a comprehensive morbidity burden diagnoses-based measure, this study showed relative underutilization in use of specialist and diagnostic services, and thus allowed for identification of inequity in health resources use, which could not be detected with less comprehensive forms of health-needs adjustments.

## Background

The link between socioeconomic status (SES) and health has long been recognized, with lower SES associated with poorer health [1,2]. With more health needs, low SES populations would be expected to consume more health services, yet accessing certain types of services is often impeded by financial and organizational barriers. Inequitable use of health services has been described even in countries with universal coverage. In In Israel, all residents are covered by mandatory health insurance, financed mainly by a progressive health tax, and provided by one of four health funds, operating as insurers and providers. Clalit Health Services is the largest (non-for-profit) health fund in Israel, with over 3.9 million enrolees (53% market share), operating services distributed throughout Israel, including 1500 primary and secondary care clinics, 14 hospitals, labs and diagnostic imaging facilities. Clalit members receive, at the point of care, free primary care and hospitalization services. Specialty care and imaging services incur a copayment. Persons of low SES (who receive social security entitlements) receive a complete or partial waiver for these copayments, depending on their social security entitlement status. Despite universal coverage and copayment waivers, previous Israeli studies have shown that disadvantaged groups face more barriers to specialty care than the rest of the population [3,4].

Studies from other developed countries have demonstrated that persons of low economic status utilize less specialist services than their more affluent counterparts [5-8], yet findings on pro-rich specialty care use also exist [9]. Primary care is generally shown to be equitably distributed in universal coverage health care systems [6,10], however inequitable primary care use is also reported [11]. These inconsistent findings may reflect actual variations in patterns of SES and health care use, yet they may be a result of differences in measurement of health needs [12].

Various measures of morbidity are used for case-mix adjustments in the health care inequity literature. The most commonly used measures include survey-based health status measures [6,7], comorbidity indices such as the Charlson comorbidity index [13], simple morbidity counts [14], or diagnoses-based morbidity measures [15]. Studies show that the use of a comprehensive survey based health-needs adjustment measure affects the degree to which equitable resource use can be assessed [6,7]. A Dutch study on SES utilization differences has shown that by controlling for health status (using a survey-based measure), differences by SES changed markedly for all health services analyzed, as compared to a basic age-gender adjustment method [16]. Yet, survey-derived measures are not easily collectable. Moreover, patient self-reported health status has been shown to differ between population sub-groups [17,18]. The relative value of different adjustment measures that are based on readily available morbidity data (from electronic medical records or administrative data bases), such as the Charlson index, or comprehensive diagnoses-based morbidity measures, has not been previously assessed.

The aim of this study was to examine the degree to which adjustment for morbidity using a diagnoses-based morbidity measure-based on tools of the Johns Hopkins University Adjusted Clinical Groups^® ^(ACGs) [19-22], explains differences in health care use by socioeconomic status, better than other commonly used health needs measures. We compared models of SES utilization differences, adjusting for overall morbidity burden (using the ACG system), with models that used age and gender only, or comorbidity (using the Charlson index).

## Methods

### Sample and setting

A representative sample of about 10% of all members of Clalit during the entire year of 2009, including persons born or those who have died during the year, was selected. All identifying data were removed. Data on diagnoses were retrieved from two main sources: hospitalization and community physician visits, which represent the entire experience each patient had within the Clalit health care system. Diagnoses are routinely electronically recorded during all medical encounters for clinical and care management (non-billing) purposes, and are then stored in a central data warehouse. Therefore, the entire morbidity profile of each individual was captured. Clalit's institutional ethics committee authorized the study.

### Morbidity groups

The JHU-ACG System takes into consideration the entire health status of a person-based on individual diagnoses and, more importantly, combinations of these diagnoses, and then classifies the population into homogeneous categories in terms of the overall illness burden [19,20]. The building blocks of the ACG system are diagnostic codes, age and gender. Each diagnoses assigned to a person during a predetermined period (usually a year) is coded into one of 32 groups-the Aggregated Diagnostic Groups (ADGs). The ADG classification (performed by the software grouper mechanism) is based on the duration, severity, diagnostic certainty, etiology, and need for specialty care that is associated with each diagnosis [19]. In this study, the ACG^® ^Case-Mix System, Version 9.1 software was used [23].

Before applying ADGs to examine socioeconomic differences in utilization within Clalit, the applicability of the ACG system to Israeli data was tested. We performed assessments of the degree to which distribution of the Clalit sample to ADGs is similar to their distributions in other countries, and we tested how well ADGs explain resource use within Clalit, thereby making the conclusions about SES valid. We compared the distribution of ADGs in Clalit to data from the Untied States (US) and Spain, as in both these countries extensive research and validation of the ACG system has been conducted and since they reflect a diverse set of health system types. The Spanish sample is from a study of Orueta and colleagues [22] on explanation of utilization of primary care in Spain based on information registered in medical records. US data came from a sample of 3 million commercially insured persons under the age of 65 from 85 geographically diverse health plans (Midwest, 35%, Northeast, 21%, South, 31%, West, 13%). Data were obtained from the PharMetrics Patient-Centric Data-base, of health plans that submit data in exchange for comparative benchmarks. A sub-sample of Clalit enrolees under the age of 65 (N = 351,558), was used for the US sample comparisons. The distribution of ADGs was compared using Pearson's r [24]. We excluded categories that had less than 100 persons (0.01%) due to small cell size considerations.

As part of the validation tests, examination of the degree to which morbidity, as measured by the ACG system, is associated with health care resource use was tested and compared to other health needs measures. The adjusted R^2 ^of several multiple linear regression models were calculated. The dependent variables were: number of primary care encounters, number of specialist visits, performance of diagnostic imaging tests, and number of hospitalizations for each person during the 12-month study period. Independent variables included age and gender, morbidity groups-the 32 ADG dummy variables, or the Charlson Comorbidity Index (CCI) [25]. The CCI was originally developed as a means of classifying the number and seriousness of about 20 chronic conditions to predict 1-year mortality based on diagnoses from medical charts, however, it is widely used to adjust for comorbidity in explanations of health care use [26,27]. Data on chronic conditions were obtained from the Clalit's Chronic Disease Registry (CCDR), which aligns information from electronic medical records of physician visits and hospitalizations, as well as data on prescription drugs and information from diagnostic and lab tests. The registry's classification is verified by individual physicians, that request changes in their patients' chronic illness status in cases of misclassification [28].

### Differences by socioeconomic status

To assess differences in health care utilization we used a sub-sample of adults aged 18 or older (N = 279,241). We examined the odds of having an above average number of primary care visits, specialist visits, or diagnostic tests performed, or having one or more hospitalization for persons holding a social security waiver versus all other adults. A social security waiver is provided to about 10% of the Israeli population receiving supplemental income (by proof of entitlement) and to those with a disability benefit, and provides a waiver of co-payments for specialty visits and diagnostic tests, and is commonly used as a marker of poor economic status in Israeli health care reports [29]. We categorized the waiver variable into two groups: those holding a waiver due to economic entitlement (1) versus all others (0), including non-waiver holders and those holding the waiver due to disability.

Analyses were conducted using three types of logistic regression models: Models A, control for age and gender only; Models B, control for age, gender and the comorbidity (using the CCI); and Models C control for age, gender, and morbidity burden (measured by ADGs). Model fit was assessed using the Akaike Information Criterion (AIC), indicating better fit for smaller versus larger AIC values, and the area under the Receiving Operator Characteristic (ROC) curve, indicating better fit as the value nears one (score ranges 0.5-1) [30].

To account for the fact that entitlement to a social security waiver is not a direct measure of socioeconomic status we conducted sensitivity analyses using two other proxy measures of SES-having supplementary private insurance (in addition to the mandatory tax-based insurance) and belonging to a low, medium, or high socioeconomic area (as measured by the socioeconomic indicator of the Israeli Bureau of Statistics [31]). To account for nesting of patients within clinics, analysis involving the area level SES indicator was conducted using the generalized estimating equation [32]. Data analyses were performed using STATA version 10 [33].

## Results

During 2009 a total of over 6 million diagnoses from hospitalizations or community physician visits were assigned to all persons in the Clalit sample (average of 6.3 distinct diagnoses per person). On average each person was assigned 4.2 morbidity groups (ADGs) (SD 3.2, range: 0-25, of a possible 32 groups) (Table 1). Social security waiver holders (adults) had on average of about 1.5 times more morbidity groups assigned than non waiver holders (6.5 ADGs, SD: 3.8 and 4.3 ADGs, SD: 3.1 respectively). The average CCI score for the total sample and for the adults sub-sample was 1.2 and 1.6, respectively (range in each sample 0-22). About 60% of all enrolees (and about 50% of all adults) had a CCI score of zero.

**Table 1:** Sample characteristics

	Total Sample (N = 398,537)	Adult Sub-sample (N = 279,241)
Age (mean, SD)	35.3 (23.1)	45.8 (19.4)
Gender, female (N, %)	203,652 (51.0)	145,205 (52.0)
Social Security Waiver (N, %)	49,241 (12.4)	40,947 (14.6)
Charlson Comorbidity Index (mean, SD)	1.19 (2.1)	1.62 (2.3)
Number of ADGs (mean, SD)	4.2 (3.2)	4.7 (3.5)

SD: Standard Deviation

ADG: Aggregate Diagnostic Groups (of possible 32 groups).

Tests of the correlation of the overall distribution of ADGs in Clalit showed that ADGs are distributed similarly in different populations. The Pearson correlation was very similar to that of the US and the Spanish samples (r = 0.89, and 0.95 respectively).

Table 2 shows that morbidity groups (ADGs) explained the largest percent of variance or in health care resource use: 23% to 54% of the variation in primary care physician visits, specialist visits, performance of diagnostic tests, and hospitalizations. Models that adjusted for comorbidity (using the CCI) explained only 11-18% of all types of resource use. Age and gender alone explained only 5-13% of variation in use of the above health care services.

**Table 2:** Coefficients of determination (*r*2) of the multiple linear regression models explaining resource use

	Primary care visits	Specialist visits	Diagnostic tests	Hospitalizations
Age and gender	0.13	0.12	0.13	0.05
Charlson Comorbidity Index, age, gender	0.18	0.13	0.15	0.11
ADGs, age, gender	0.54	0.45	0.37	0.24

ADG: Aggregate Diagnostic Groups (dummy variables, non-mutually exclusive).

### Differences in resource use by socioeconomic status

Table 3 reports the differences in high resource use between persons holding a social-security waiver and all other adult enrolees. Adults holding a social-security waiver were about twice as likely to have an above average number of primary care visits, 30% more likely to have an above average number of specialist visits or diagnostic tests performed, and more than twice as likely to be hospitalized at least one, compared with all other adults.

**Table 3:** Percent with high service use by socioeconomic status*

	Adults with Social Security Waiver	All other adults
Above average number of primary care visits	63%	34%
Above average number of specialist visits	42%	31%
Above average number of diagnostic tests	38%	28%
One or more hospitalizations	16%	7%

* p-value from chi square tests: p < 0.001 for all comparisons

Table 4 reports the Odds Ratios (OR) and confidence intervals (CI) for having above average resource use in persons with a social security waiver compared to all other adults. Controlling for age and gender only or for comorbidity (using the CCI), persons of low socioeconomic status (i.e., those holding a waiver) were significantly more likely to have above average number of primary care visits, specialist visits, diagnostic tests performed, and one or more hospitalization. Controlling for morbidity burden (using ADGs), compared to all other adults, waiver holders were less likely to have an above average number of specialist visits or an above average number of diagnostic tests performed. In all morbidity burden models (Models C), the likelihood of having an above average resource use for each type of service examined was reduced by 17-35% relative to the age and gender only models (Models A).

**Table 4:** Socioeconomic class and high service use: odds-ratios for alternative morbidity adjustment models

*Adults with Social Security Waiver (vs. all other adults)*		Above average number of primary care visits	Above average number of specialist visits	Above average number of diagnostic tests	One or more hospitalizations
Model A: Adjusting for age and gender					
Odds ratio (95% CI)		1.92 (1.87-1.97)	1.13 (1.10-1.57)	1.11 (1.08-1.37)	1.67 (1.62-1.73)

Model B: Adjusting for age, gender and the Charlson Comorbidity Index					
Odds ratio (95% CI)		1.62 (1.58-1.66)	1.04 (1.01-1.06)	1.05 (1.03-1.08)	1.38 (1.33-1.42)

Model C: Adjusting for age, gender, and morbidity using ADG categories					
Odds ratio (95% CI)		1.64 (1.60-1.69)	0.95 (0.94-0.99)	0.91 (0.86-0.96)	1.24 (1.20-1.29)

ADG: Aggregate Diagnostic Groups (dummy variables, non-mutually exclusive).

CI: Confidence Interval

Tests of model fit (area under the ROC curve or AIC tests) indicated a better fit of all morbidity burden models (Models C; area under the ROC curve: between 0.72-0.86, indicating good-very good fit) than models controlling for age and gender only (Models A; area under the ROC curve: between 0.66-0.74, indicating a poor-good fit) or the comorbidity (CCI) models (Models B; area under the ROC curve: between 0.67-0.77, indicating a poor-good fit) [23] (Table 5).

**Table 5:** Socioeconomic class and high service use: predictive accuracy of alternative morbidity models

*Adults with Social Security Waiver (vs. all other adults)*		Above average number of primary care visits	Above average number of specialist visits	Above average number of diagnostic tests	One or more hospitalizations
Model A: Adjusting for age and gender					
Area under the ROC curve		0.74	0.66	0.66	0.67
AIC		315900	331209	262971	150883

Model B: Adjusting for age, gender and the Charlson Comorbidity Index					
Area under the ROC curve		0.77	0.67	0.67	0.72
AIC		307183	329009	262267	142501

Model C: Adjusting for age, gender, and morbidity using ADG categories					
Area under the ROC curve		0.85	0.77	0.72	0.86
AIC		254608	290254	247195	117215

ROC: Receiver Operating Characteristics

ADG: Aggregate Diagnostic Groups (dummy variables, non-mutually exclusive).

AIC: Akaike Information Criteria

Analysis conducted with the two other SES measures (having supplemental insurance or area level SES) showed similar results-i.e., controlling for age, gender, and morbidity burden, the more socially deprived were significantly more likely to have an above average number of primary care visits or one or more hospitalizations, and significantly less likely to have an above average number of specialist visits or diagnostic tests performed, than those with supplemental private insurance or higher SES (data not shown). All models that accounted for overall morbidity burden resulted in a better fit than adjustments using the comorbidity measure or age and gender only.

## Discussion

Our findings show that accounting for overall morbidity levels with a comprehensive measure based on the combination of all types of diagnoses, inequity in various types of health care resource use could be identified. Persons of low economic status were more likely than those of higher economic status to be high users of primary care services, more likely to be hospitalized at least once during the 12-month study period, and were less likely to be high users of specialty care and diagnostic imaging services.

Comparisons between models that used different adjustment measures showed that morbidity burden provides a better explanation of differential resource use than other commonly used measures. Adjustments using age and gender or the Charlson index overestimated the difference in utilization between persons from low and high SES. Moreover, adjusting for age and gender or the Charlson index misleadingly showed that persons of low SES are more likely to have higher utilization of specialists and diagnostic services. Adjustments using the morbidity burden measure (ADGs) revealed a reverse relationship, more accurately explaining differences in resource use (as shown by the tests of model fit).

Other countries and health systems with universal coverage face similar challenges-differences in health care use by SES are reported, with reports on pro-rich inequity in specialty care use [5-8] and in performance of diagnostic imaging tests [15,34]. Studies on primary care use by SES are mixed, with some showing an equitable (i.e., needs-based) distribution across SES groups [6,10], while others support the findings reported here, showing that person of poorer economic status use more primary care services than their richer counterparts [35]. Higher rates of hospitalizations of those from lower SES were also found by others [14]. Our study supports these findings, and adds to current knowledge by showing that the choice of health-need adjustment measure can affect the magnitude and accuracy of identification of gaps in service use.

Countries and health care systems differ in benefit design, patient-cost-sharing, and the role of private insurance, and the reasons and magnitude of inequalities vary. Yet, common findings on utilization inequity in universal coverage systems suggest that implementation of universal coverage principles deserves further consideration. A possible explanation for the inequitable use of health services reported here is that, as reported also by others, persons of low SES face non-financial barriers to health service use [3,4]. These barriers may include poorer availability of services, cultural and language gaps that may affect minorities, who constitute large percentages of low SES populations, or differences in preferences. Future research is required to test whether differences in use reflect the level of needed care by persons from diverse SES groups (i.e., whether underutilization or overutilization exists) and to assess the degree to which inequity reduction programs succeed in minimizing unwarranted gaps.

A potential criticism on the use of diagnoses-based measures, such as ADGs, is that they may be biased due to their reliance on data registered during patient visits, and thus non-clinically measured aspect of health and underutilization may affect the completeness of data. A recent Canadian study addressed this potential shortcoming by examining the contribution of survey-derived indicators of health status to explanatory models of physician service use based on morbidity adjustment using the ACG system [36]. Adjustments for health status in that study did not contribute significantly to models on the basis of the diagnoses-based ACG measures. As availability of survey data, in comparison to routinely collected administrative and clinical data, may be limited, it is important to acknowledge the benefits of using diagnoses-based measures for planning, reimbursement, and research.

Assessing healthcare utilization patterns among low SES groups is a key step in planning health inequity-reduction strategies in healthcare organizations. To reduce inequities in health and in the delivery of health care, Clalit has laid out in 2008 an organization-wide strategic plan that addresses health care workforce, quality of care and utilization differences between low and high SES groups [37]. Examination of differences in use of health care services, based on robust measures of need, as reported here, can direct organizational efforts by suggesting areas of potential inequitable access to care.

Diagnoses-based morbidity measures, which classify the population according to diagnoses from all medical encounters, are increasingly being used by health care organizations worldwide for various applications, including equitable allocation of resources, assessments of providers' performance, care management, and for research and evaluation [28,38,39]. Clalit Health Services, mainly due to historical reasons [40], has a significant overrepresentation of underprivileged populations-low socioeconomic groups, minorities, new immigrants, and persons with disabilities [41]. Clalit has developed in recent years various case-mix tools for medical, economic and administrative purposes. A recent study has demonstrated the feasibility and validity of using ACGs in Clalit [42]. Our cross-national comparisons, as well as studies from other countries [43-45], show that ACGs provide robust classification of morbidity across different countries with markedly different health care systems. Moreover, our results show that ACGs explain a large percent of resource use at Clalit, similar to reports from other countries; ACGs have been shown to explain 53-59% of the variation in primary care visits in Spain [22], 41-58% of variation in ambulatory visits in Taiwan [46], and 32-59% of ambulatory visits in the US [19].

A potential limitation of any diagnoses-based system is the quality of the diagnostic coding. The accuracy of the diagnoses has not been systematically estimated here, however, the similarity between the ACGs' distributions in different countries suggests that coding is not a major limitation in Clalit. Additionally, the JHU-ACG system is relatively robust and minor differences in coding do not necessarily affect the system's groupings, which are based on types of diagnoses and health states and not on specific diagnoses and diseases.

Another limitation is that our measure of low SES is only a proxy measure and does not incorporate important information on education, income and wealth components [47]. To address this we have examined different proxies for individual SES, all leading to the same results regarding the direction, magnitude and significance of difference between those with lower versus higher SES.

Additionally, our results may not be representative of differences in utilization between socioeconomic groups in other countries. Health care systems world-wide differ in benefit design, patient-cost-sharing, and the role of private insurance. Yet, our main finding, i.e., that adjustment for morbidity using a robust diagnoses-based measure allows for a more accurate assessment of inequity in resource use is of relevance to other countries and health systems. Finally, this study only examines how well morbidity, as measured by the ACG system, explains actual service use, rather than what ought to have been provided based on patients' needs. Future studies are needed to test whether adjustment for additional factors that define health care need (e.g., proximity to healthcare facilities or patient care seeking behaviours) provide additional insight into the reasons for differences in healthcare use.

## Conclusions

Relative to all other enrolees, persons of low socioeconomic status were less likely to be high users of specialty care and imaging services, a phenomenon identified only with the use of a robust health-needs measure. Using routinely recorded data from electronic medical records, the JHU-ACG System is a feasible and valid method to account for the morbidity level of an Israeli population, strongly associated with different types of healthcare services utilization. Accurate adjustment to morbidity level afforded by this system, allows new insights on differences in healthcare services utilization between population sub-groups.

With the launching of a new inequity reduction strategy within Clalit, accurate assessment of these differences is of considerable importance, as assessment of inequalities in health and health care, care management and resource allocation can be performed more precisely and fairly. Further research is needed to test these differences in other Israeli populations as well as in other countries and to better understand the possible contribution of differences in utilization by SES to health outcomes.

## Competing interests

The ACG Systems is commercially distributed under license with The Johns Hopkins University, which holds the copyright to the ACG System. Johns Hopkins University benefits from the sale of this software. The majority of these royalties is used toward supporting ongoing development work of the ACG System, including the research presented in this article.

## Authors' contributions

ES and RDB designed the study, performed the statistical analysis and drafted the manuscript. CA, KK, and JW provided insight into the concept and design of the study and revised the manuscript critically for important intellectual content. All authors read and approved the final version of the manuscript.

## Pre-publication history

The pre-publication history for this paper can be accessed here:

http://www.biomedcentral.com/1471-2458/11/609/prepub
